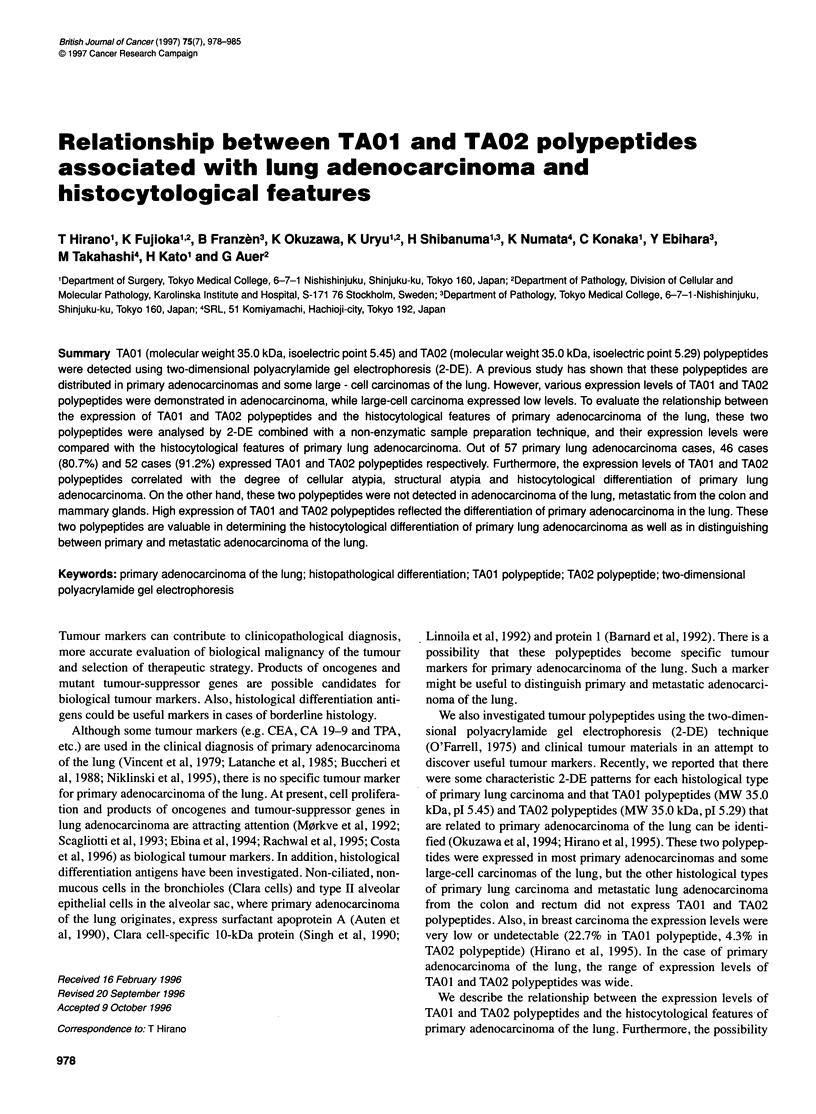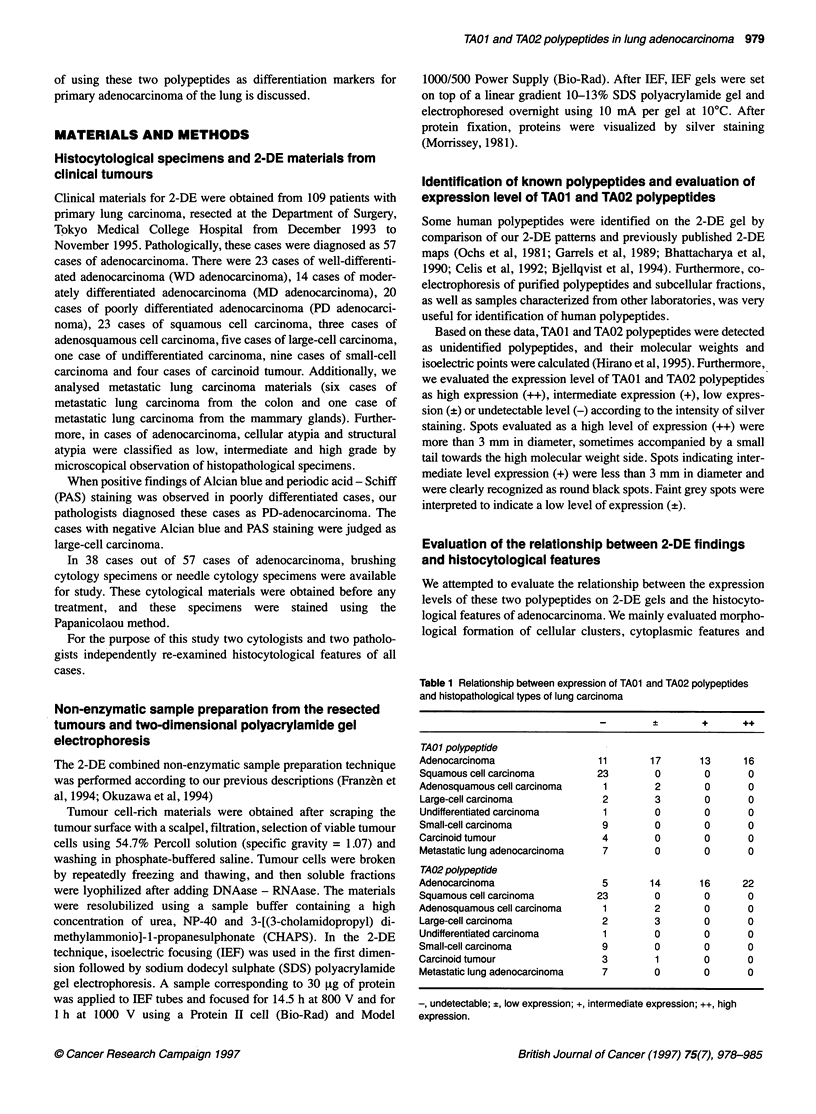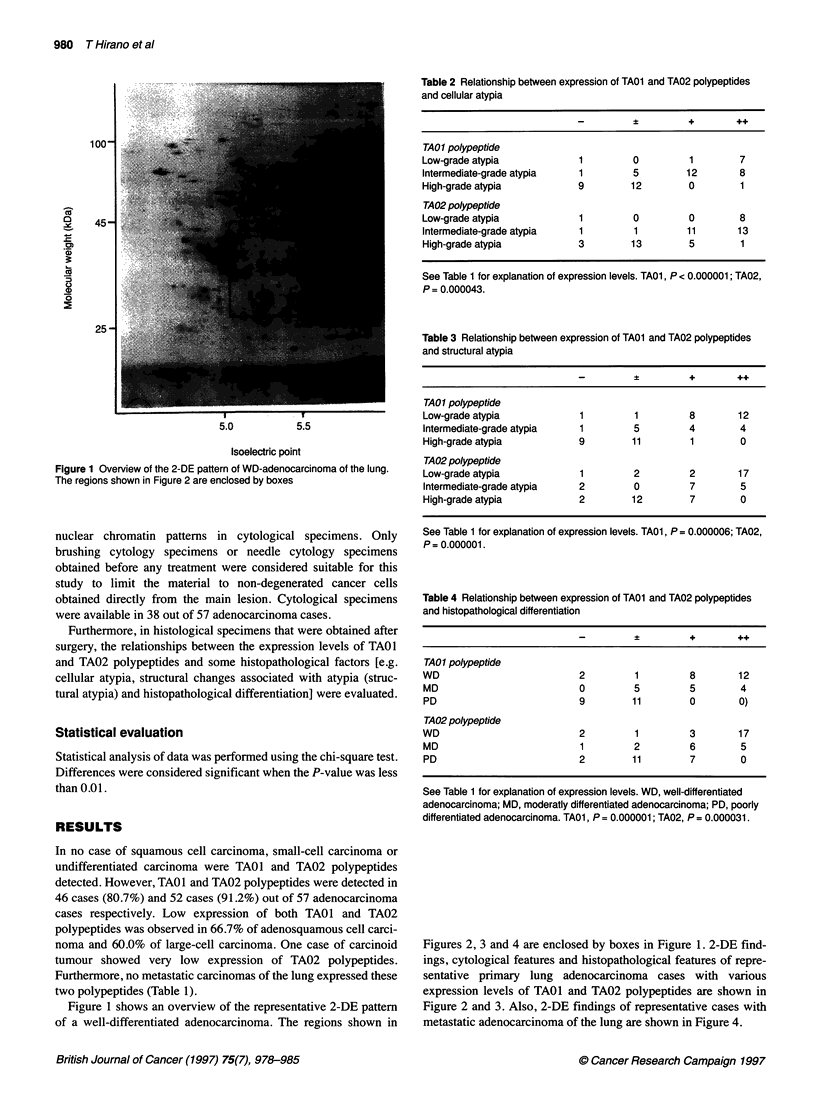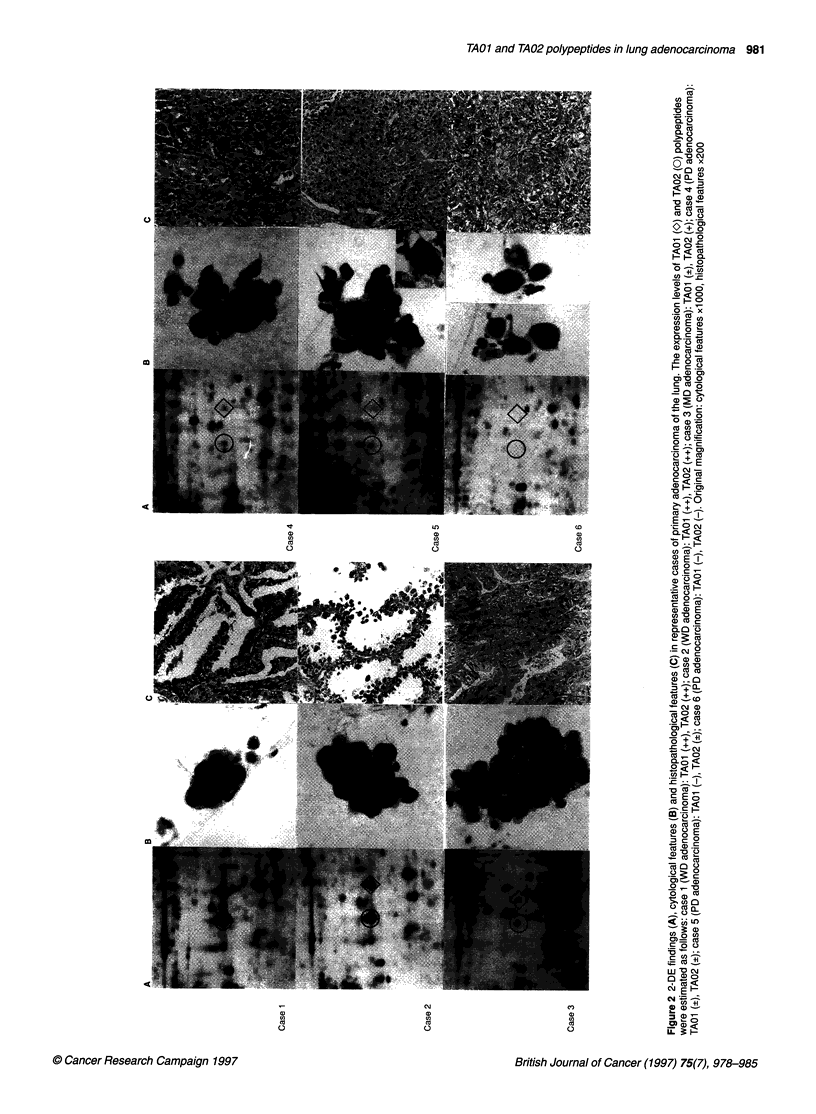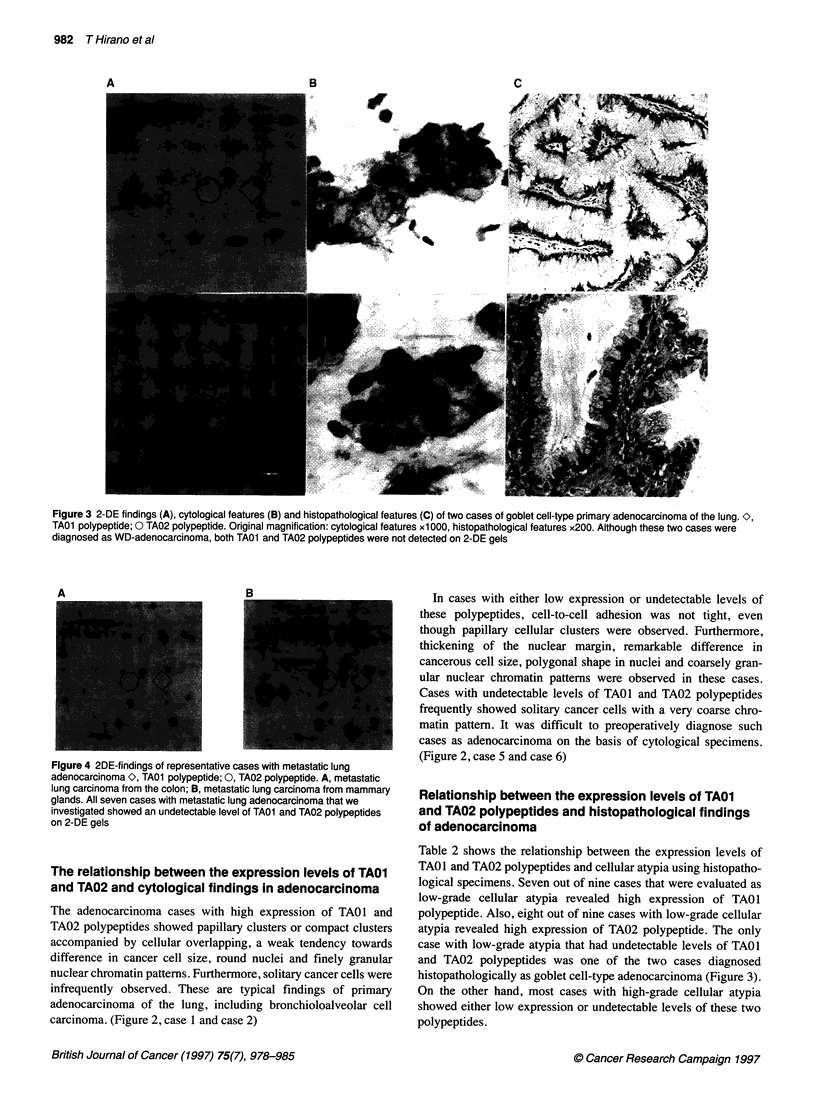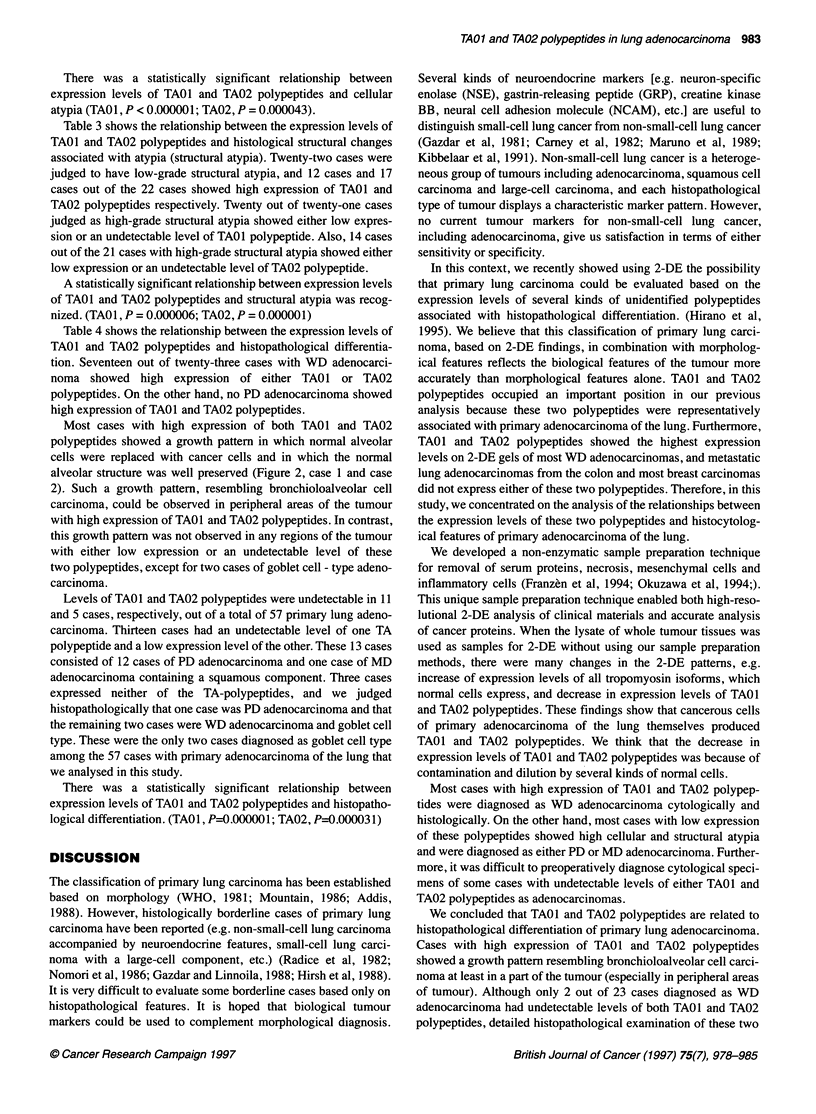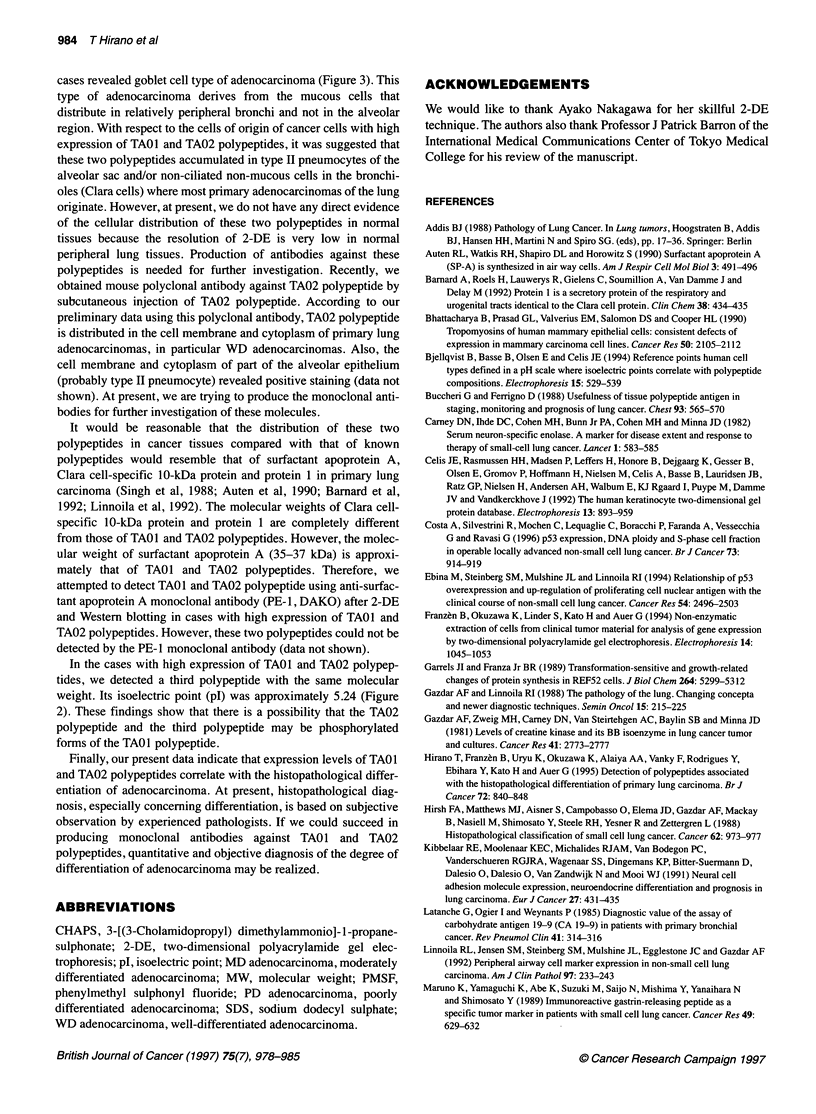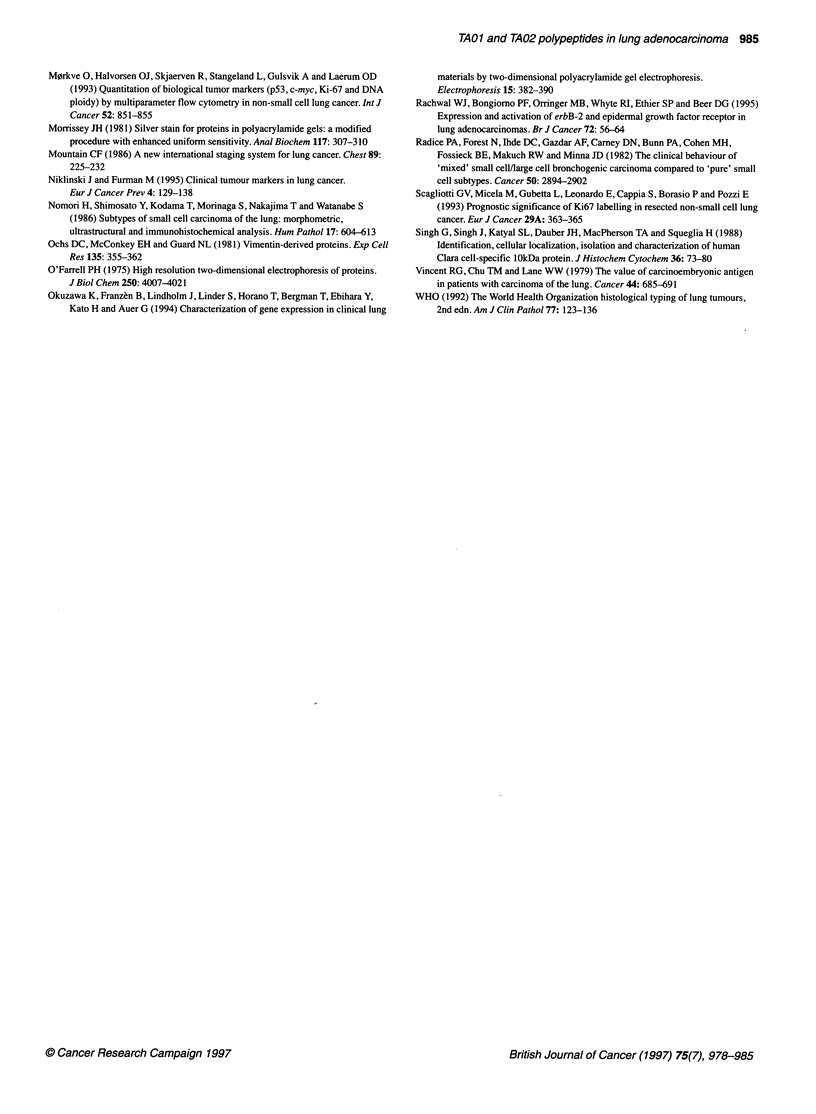# Relationship between TA01 and TA02 polypeptides associated with lung adenocarcinoma and histocytological features.

**DOI:** 10.1038/bjc.1997.169

**Published:** 1997

**Authors:** T. Hirano, K. Fujioka, B. FranzÃ¨n, K. Okuzawa, K. Uryu, H. Shibanuma, K. Numata, C. Konaka, Y. Ebihara, M. Takahashi, H. Kato, G. Auer

**Affiliations:** Department of Surgery, Tokyo Medical College, Shinjuku-ku, Japan.

## Abstract

**Images:**


British Journal of Cancer (1997) 75(7), 978–985
© 1997 Cancer Research Campaign

### Relationship between TA01 and TA02 polypeptides associated with lung adenocarcinoma and histocytological features

T Hirano[1], K Fujioka[1,2], B Franzèn[3], K Okuzawa, K Uryu[1,2], H Shibanuma[1,3], K Numata[4], C Konaka[1], Y Ebihara[3], M Takahashi[4], H Kato[1] and G Auer[2]

[1]Department of Surgery, Tokyo Medical College, 6–7–1 Nishishinjuku, Shinjuku-ku, Tokyo 160, Japan; [2]Department of Pathology, Division of Cellular and Molecular Pathology, Karolinska Institute and Hospital, S-171 76 Stockholm, Sweden; [3]Department of Pathology, Tokyo Medical College, 6–7–1-Nishishinjuku, Shinjuku-ku, Tokyo 160, Japan; [4]SRL, 51 Komiyamachi, Hachioji-city, Tokyo 192, Japan

**Summary** TA01 (molecular weight 35.0 kDa, isoelectric point 5.45) and TA02 (molecular weight 35.0 kDa, isoelectric point 5.29) polypeptides were detected using two-dimensional polyacrylamide gel electrophoresis (2-DE). A previous study has shown that these polypeptides are distributed in primary adenocarcinomas and some large - cell carcinomas of the lung. However, various expression levels of TA01 and TA02 polypeptides were demonstrated in adenocarcinoma, while large-cell carcinoma expressed low levels. To evaluate the relationship between the expression of TA01 and TA02 polypeptides and the histocytological features of primary adenocarcinoma of the lung, these two polypeptides were analysed by 2-DE combined with a non-enzymatic sample preparation technique, and their expression levels were compared with the histocytological features of primary lung adenocarcinoma. Out of 57 primary lung adenocarcinoma cases, 46 cases (80.7%) and 52 cases (91.2%) expressed TA01 and TA02 polypeptides respectively. Furthermore, the expression levels of TA01 and TA02 polypeptides correlated with the degree of cellular atypia, structural atypia and histocytological differentiation of primary lung adenocarcinoma. On the other hand, these two polypeptides were not detected in adenocarcinoma of the lung, metastatic from the colon and mammary glands. High expression of TA01 and TA02 polypeptides reflected the differentiation of primary adenocarcinoma in the lung. These two polypeptides are valuable in determining the histocytological differentiation of primary lung adenocarcinoma as well as in distinguishing between primary and metastatic adenocarcinoma of the lung.

**Keywords:** primary adenocarcinoma of the lung; histopathological differentiation; TA01 polypeptide; TA02 polypeptide; two-dimensional polyacrylamide gel electrophoresis

Tumour markers can contribute to clinicopathological diagnosis, more accurate evaluation of biological malignancy of the tumour and selection of therapeutic strategy. Products of oncogenes and mutant tumour-suppressor genes are possible candidates for biological tumour markers. Also, histological differentiation antigens could be useful markers in cases of borderline histology.

Although some tumour markers (e.g. CEA, CA 19–9 and TPA, etc.) are used in the clinical diagnosis of primary adenocarcinoma of the lung (Vincent et al, 1979; Latanche et al, 1985; Buccheri et al, 1988; Niklinski et al, 1995), there is no specific tumour marker for primary adenocarcinoma of the lung. At present, cell proliferation and products of oncogenes and tumour-suppressor genes in lung adenocarcinoma are attracting attention (Mørkve et al, 1992; Scagliotti et al, 1993; Ebina et al, 1994; Rachwal et al, 1995; Costa et al, 1996) as biological tumour markers. In addition, histological differentiation antigens have been investigated. Non-ciliated, non-mucous cells in the bronchioles (Clara cells) and type II alveolar epithelial cells in the alveolar sac, where primary adenocarcinoma of the lung originates, express surfactant apoprotein A (Auten et al, 1990), Clara cell-specific 10-kDa protein (Singh et al, 1990; Linnoila et al, 1992) and protein 1 (Barnard et al, 1992). There is a possibility that these polypeptides become specific tumour markers for primary adenocarcinoma of the lung. Such a marker might be useful to distinguish primary and metastatic adenocarcinoma of the lung.

We also investigated tumour polypeptides using the two-dimensional polyacrylamide gel electrophoresis (2-DE) technique (O'Farrell, 1975) and clinical tumour materials in an attempt to discover useful tumour markers. Recently, we reported that there were some characteristic 2-DE patterns for each histological type of primary lung carcinoma and that TA01 polypeptides (MW 35.0 kDa, pI 5.45) and TA02 polypeptides (MW 35.0 kDa, pI 5.29) that are related to primary adenocarcinoma of the lung can be identified (Okuzawa et al, 1994; Hirano et al, 1995). These two polypeptides were expressed in most primary adenocarcinomas and some large-cell carcinomas of the lung, but the other histological types of primary lung carcinoma and metastatic lung adenocarcinoma from the colon and rectum did not express TA01 and TA02 polypeptides. Also, in breast carcinoma the expression levels were very low or undetectable (22.7% in TA01 polypeptide, 4.3% in TA02 polypeptide) (Hirano et al, 1995). In the case of primary adenocarcinoma of the lung, the range of expression levels of TA01 and TA02 polypeptides was wide.

We describe the relationship between the expression levels of TA01 and TA02 polypeptides and the histocytological features of primary adenocarcinoma of the lung. Furthermore, the possibility

*Received 16 February 1996*
*Revised 20 September 1996*
*Accepted 9 October 1996*

*Correspondence to:* T Hirano of using these two polypeptides as differentiation markers for primary adenocarcinoma of the lung is discussed.

### MATERIALS AND METHODS

### Histocytological specimens and 2-DE materials from clinical tumours

Clinical materials for 2-DE were obtained from 109 patients with primary lung carcinoma, resected at the Department of Surgery, Tokyo Medical College Hospital from December 1993 to November 1995. Pathologically, these cases were diagnosed as 57 cases of adenocarcinoma. There were 23 cases of well-differentiated adenocarcinoma (WD adenocarcinoma), 14 cases of moderately differentiated adenocarcinoma (MD adenocarcinoma), 20 cases of poorly differentiated adenocarcinoma (PD adenocarcinoma), 23 cases of squamous cell carcinoma, three cases of adenosquamous cell carcinoma, five cases of large-cell carcinoma, one case of undifferentiated carcinoma, nine cases of small-cell carcinoma and four cases of carcinoid tumour. Additionally, we analysed metastatic lung carcinoma materials (six cases of metastatic lung carcinoma from the colon and one case of metastatic lung carcinoma from the mammary glands). Furthermore, in cases of adenocarcinoma, cellular atypia and structural atypia were classified as low, intermediate and high grade by microscopical observation of histopathological specimens.

When positive findings of Alcian blue and periodic acid – Schiff (PAS) staining was observed in poorly differentiated cases, our pathologists diagnosed these cases as PD-adenocarcinoma. The cases with negative Alcian blue and PAS staining were judged as large-cell carcinoma.

In 38 cases out of 57 cases of adenocarcinoma, brushing cytology specimens or needle cytology specimens were available for study. These cytological materials were obtained before any treatment, and these specimens were stained using the Papanicolaou method.

For the purpose of this study two cytologists and two pathologists independently re-examined histocytological features of all cases.

### Non-enzymatic sample preparation from the resected tumours and two-dimensional polyacrylamide gel electrophoresis

The 2-DE combined non-enzymatic sample preparation technique was performed according to our previous descriptions (Franzèn et al, 1994; Okuzawa et al, 1994)

Tumour cell-rich materials were obtained after scraping the tumour surface with a scalpel, filtration, selection of viable tumour cells using 54.7% Percoll solution (specific gravity = 1.07) and washing in phosphate-buffered saline. Tumour cells were broken by repeatedly freezing and thawing, and then soluble fractions were lyophilized after adding DNAase – RNAase. The materials were resolubilized using a sample buffer containing a high concentration of urea, NP-40 and 3-[(3-cholamidopropyl) dimethylammonio]-1-propanesulphonate (CHAPS). In the 2-DE technique, isoelectric focusing (IEF) was used in the first dimension followed by sodium dodecyl sulphate (SDS) polyacrylamide gel electrophoresis. A sample corresponding to 30 μg of protein was applied to IEF tubes and focused for 14.5 h at 800 V and for 1 h at 1000 V using a Protein II cell (Bio-Rad) and Model 1000/500 Power Supply (Bio-Rad). After IEF, IEF gels were set on top of a linear gradient 10–13% SDS polyacrylamide gel and electrophoresed overnight using 10 mA per gel at 10°C. After protein fixation, proteins were visualized by silver staining (Morrissey, 1981).

### Identification of known polypeptides and evaluation of expression level of TA01 and TA02 polypeptides

Some human polypeptides were identified on the 2-DE gel by comparison of our 2-DE patterns and previously published 2-DE maps (Ochs et al, 1981; Garrels et al, 1989; Bhattacharya et al, 1990; Celis et al, 1992; Bjellqvist et al, 1994). Furthermore, co-electrophoresis of purified polypeptides and subcellular fractions, as well as samples characterized from other laboratories, was very useful for identification of human polypeptides.

Based on these data, TA01 and TA02 polypeptides were detected as unidentified polypeptides, and their molecular weights and isoelectric points were calculated (Hirano et al, 1995). Furthermore, we evaluated the expression level of TA01 and TA02 polypeptides as high expression (++), intermediate expression (+), low expression (±) or undetectable level (–) according to the intensity of silver staining. Spots evaluated as a high level of expression (++) were more than 3 mm in diameter, sometimes accompanied by a small tail towards the high molecular weight side. Spots indicating intermediate level expression (+) were less than 3 mm in diameter and were clearly recognized as round black spots. Faint grey spots were interpreted to indicate a low level of expression (±).

### Evaluation of the relationship between 2-DE findings and histocytological features

We attempted to evaluate the relationship between the expression levels of these two polypeptides on 2-DE gels and the histocytological features of adenocarcinoma. We mainly evaluated morphological formation of cellular clusters, cytoplasmic features and

**Table 1** Relationship between expression of TA01 and TA02 polypeptides and histopathological types of lung carcinoma

| | – | ± | + | ++ |
|---|---|---|---|---|
| *TA01 polypeptide* | | | | |
| Adenocarcinoma | 11 | 17 | 13 | 16 |
| Squamous cell carcinoma | 23 | 0 | 0 | 0 |
| Adenosquamous cell carcinoma | 1 | 2 | 0 | 0 |
| Large-cell carcinoma | 2 | 3 | 0 | 0 |
| Undifferentiated carcinoma | 1 | 0 | 0 | 0 |
| Small-cell carcinoma | 9 | 0 | 0 | 0 |
| Carcinoid tumour | 4 | 0 | 0 | 0 |
| Metastatic lung adenocarcinoma | 7 | 0 | 0 | 0 |
| *TA02 polypeptide* | | | | |
| Adenocarcinoma | 5 | 14 | 16 | 22 |
| Squamous cell carcinoma | 23 | 0 | 0 | 0 |
| Adenosquamous cell carcinoma | 1 | 2 | 0 | 0 |
| Large-cell carcinoma | 2 | 3 | 0 | 0 |
| Undifferentiated carcinoma | 1 | 0 | 0 | 0 |
| Small-cell carcinoma | 9 | 0 | 0 | 0 |
| Carcinoid tumour | 3 | 1 | 0 | 0 |
| Metastatic lung adenocarcinoma | 7 | 0 | 0 | 0 |

–, undetectable; ±, low expression; +, intermediate expression; ++, high expression.

© Cancer Research Campaign 1997 

**Figure 1** Overview of the 2-DE pattern of WD-adenocarcinoma of the lung. The regions shown in Figure 2 are enclosed by boxes nuclear chromatin patterns in cytological specimens. Only brushing cytology specimens or needle cytology specimens obtained before any treatment were considered suitable for this study to limit the material to non-degenerated cancer cells obtained directly from the main lesion. Cytological specimens were available in 38 out of 57 adenocarcinoma cases.

Furthermore, in histological specimens that were obtained after surgery, the relationships between the expression levels of TA01 and TA02 polypeptides and some histopathological factors [e.g. cellular atypia, structural changes associated with atypia (structural atypia) and histopathological differentiation] were evaluated.

### Statistical evaluation

Statistical analysis of data was performed using the chi-square test. Differences were considered significant when the *P*-value was less than 0.01.

### RESULTS

In no case of squamous cell carcinoma, small-cell carcinoma or undifferentiated carcinoma were TA01 and TA02 polypeptides detected. However, TA01 and TA02 polypeptides were detected in 46 cases (80.7%) and 52 cases (91.2%) out of 57 adenocarcinoma cases respectively. Low expression of both TA01 and TA02 polypeptides was observed in 66.7% of adenosquamous cell carcinoma and 60.0% of large-cell carcinoma. One case of carcinoid tumour showed very low expression of TA02 polypeptides. Furthermore, no metastatic carcinomas of the lung expressed these two polypeptides (Table 1).

Figure 1 shows an overview of the representative 2-DE pattern of a well-differentiated adenocarcinoma. The regions shown in

**Table 2** Relationship between expression of TA01 and TA02 polypeptides and cellular atypia

| | – | ± | + | ++ |
|---|---|---|---|---|
| *TA01 polypeptide* | | | | |
| Low-grade atypia | 1 | 0 | 1 | 7 |
| Intermediate-grade atypia | 1 | 5 | 12 | 8 |
| High-grade atypia | 9 | 12 | 0 | 1 |
| *TA02 polypeptide* | | | | |
| Low-grade atypia | 1 | 0 | 0 | 8 |
| Intermediate-grade atypia | 1 | 1 | 11 | 13 |
| High-grade atypia | 3 | 13 | 5 | 1 |

See Table 1 for explanation of expression levels. TA01, $P < 0.000001$; TA02, $P = 0.000043$.

**Table 3** Relationship between expression of TA01 and TA02 polypeptides and structural atypia

| | – | ± | + | ++ |
|---|---|---|---|---|
| *TA01 polypeptide* | | | | |
| Low-grade atypia | 1 | 1 | 8 | 12 |
| Intermediate-grade atypia | 1 | 5 | 4 | 4 |
| High-grade atypia | 9 | 11 | 1 | 0 |
| *TA02 polypeptide* | | | | |
| Low-grade atypia | 1 | 2 | 2 | 17 |
| Intermediate-grade atypia | 2 | 0 | 7 | 5 |
| High-grade atypia | 2 | 12 | 7 | 0 |

See Table 1 for explanation of expression levels. TA01, $P = 0.000006$; TA02, $P = 0.000001$.

**Table 4** Relationship between expression of TA01 and TA02 polypeptides and histopathological differentiation

| | – | ± | + | ++ |
|---|---|---|---|---|
| *TA01 polypeptide* | | | | |
| WD | 2 | 1 | 8 | 12 |
| MD | 0 | 5 | 5 | 4 |
| PD | 9 | 11 | 0 | 0) |
| *TA02 polypeptide* | | | | |
| WD | 2 | 1 | 3 | 17 |
| MD | 1 | 2 | 6 | 5 |
| PD | 2 | 11 | 7 | 0 |

See Table 1 for explanation of expression levels. WD, well-differentiated adenocarcinoma; MD, moderatly differentiated adenocarcinoma; PD, poorly differentiated adenocarcinoma. TA01, $P = 0.000001$; TA02, $P = 0.000031$.

Figures 2, 3 and 4 are enclosed by boxes in Figure 1. 2-DE findings, cytological features and histopathological features of representative primary lung adenocarcinoma cases with various expression levels of TA01 and TA02 polypeptides are shown in Figure 2 and 3. Also, 2-DE findings of representative cases with metastatic adenocarcinoma of the lung are shown in Figure 4.

© Cancer Research Campaign 1997

© Cancer Research Campaign 1997

**Figure 3** 2-DE findings (**A**), cytological features (**B**) and histopathological features (**C**) of two cases of goblet cell-type primary adenocarcinoma of the lung. ◇, TA01 polypeptide; ○ TA02 polypeptide. Original magnification: cytological features ×1000, histopathological features ×200. Although these two cases were diagnosed as WD-adenocarcinoma, both TA01 and TA02 polypeptides were not detected on 2-DE gels

**Figure 4** 2DE-findings of representative cases with metastatic lung adenocarcinoma ◇, TA01 polypeptide; ○, TA02 polypeptide. **A**, metastatic lung carcinoma from the colon; **B**, metastatic lung carcinoma from mammary glands. All seven cases with metastatic lung adenocarcinoma that we investigated showed an undetectable level of TA01 and TA02 polypeptides on 2-DE gels

### The relationship between the expression levels of TA01 and TA02 and cytological findings in adenocarcinoma

The adenocarcinoma cases with high expression of TA01 and TA02 polypeptides showed papillary clusters or compact clusters accompanied by cellular overlapping, a weak tendency towards difference in cancer cell size, round nuclei and finely granular nuclear chromatin patterns. Furthermore, solitary cancer cells were infrequently observed. These are typical findings of primary adenocarcinoma of the lung, including bronchioloalveolar cell carcinoma. (Figure 2, case 1 and case 2)

In cases with either low expression or undetectable levels of these polypeptides, cell-to-cell adhesion was not tight, even though papillary cellular clusters were observed. Furthermore, thickening of the nuclear margin, remarkable difference in cancerous cell size, polygonal shape in nuclei and coarsely granular nuclear chromatin patterns were observed in these cases. Cases with undetectable levels of TA01 and TA02 polypeptides frequently showed solitary cancer cells with a very coarse chromatin pattern. It was difficult to preoperatively diagnose such cases as adenocarcinoma on the basis of cytological specimens. (Figure 2, case 5 and case 6)

### Relationship between the expression levels of TA01 and TA02 polypeptides and histopathological findings of adenocarcinoma

Table 2 shows the relationship between the expression levels of TA01 and TA02 polypeptides and cellular atypia using histopathological specimens. Seven out of nine cases that were evaluated as low-grade cellular atypia revealed high expression of TA01 polypeptide. Also, eight out of nine cases with low-grade cellular atypia revealed high expression of TA02 polypeptide. The only case with low-grade atypia that had undetectable levels of TA01 and TA02 polypeptides was one of the two cases diagnosed histopathologically as goblet cell-type adenocarcinoma (Figure 3). On the other hand, most cases with high-grade cellular atypia showed either low expression or undetectable levels of these two polypeptides.

© Cancer Research Campaign 1997

There was a statistically significant relationship between expression levels of TA01 and TA02 polypeptides and cellular atypia (TA01, $P < 0.000001$; TA02, $P = 0.000043$).

Table 3 shows the relationship between the expression levels of TA01 and TA02 polypeptides and histological structural changes associated with atypia (structural atypia). Twenty-two cases were judged to have low-grade structural atypia, and 12 cases and 17 cases out of the 22 cases showed high expression of TA01 and TA02 polypeptides respectively. Twenty out of twenty-one cases judged as high-grade structural atypia showed either low expression or an undetectable level of TA01 polypeptide. Also, 14 cases out of the 21 cases with high-grade structural atypia showed either low expression or an undetectable level of TA02 polypeptide.

A statistically significant relationship between expression levels of TA01 and TA02 polypeptides and structural atypia was recognized. (TA01, $P = 0.000006$; TA02, $P = 0.000001$)

Table 4 shows the relationship between the expression levels of TA01 and TA02 polypeptides and histopathological differentiation. Seventeen out of twenty-three cases with WD adenocarcinoma showed high expression of either TA01 or TA02 polypeptides. On the other hand, no PD adenocarcinoma showed high expression of TA01 and TA02 polypeptides.

Most cases with high expression of both TA01 and TA02 polypeptides showed a growth pattern in which normal alveolar cells were replaced with cancer cells and in which the normal alveolar structure was well preserved (Figure 2, case 1 and case 2). Such a growth pattern, resembling bronchioloalveolar cell carcinoma, could be observed in peripheral areas of the tumour with high expression of TA01 and TA02 polypeptides. In contrast, this growth pattern was not observed in any regions of the tumour with either low expression or an undetectable level of these two polypeptides, except for two cases of goblet cell - type adenocarcinoma.

Levels of TA01 and TA02 polypeptides were undetectable in 11 and 5 cases, respectively, out of a total of 57 primary lung adenocarcinoma. Thirteen cases had an undetectable level of one TA polypeptide and a low expression level of the other. These 13 cases consisted of 12 cases of PD adenocarcinoma and one case of MD adenocarcinoma containing a squamous component. Three cases expressed neither of the TA-polypeptides, and we judged histopathologically that one case was PD adenocarcinoma and that the remaining two cases were WD adenocarcinoma and goblet cell type. These were the only two cases diagnosed as goblet cell type among the 57 cases with primary adenocarcinoma of the lung that we analysed in this study.

There was a statistically significant relationship between expression levels of TA01 and TA02 polypeptides and histopathological differentiation. (TA01, $P$=0.000001; TA02, $P$=0.000031)

### DISCUSSION

The classification of primary lung carcinoma has been established based on morphology (WHO, 1981; Mountain, 1986; Addis, 1988). However, histologically borderline cases of primary lung carcinoma have been reported (e.g. non-small-cell lung carcinoma accompanied by neuroendocrine features, small-cell lung carcinoma with a large-cell component, etc.) (Radice et al, 1982; Nomori et al, 1986; Gazdar and Linnoila, 1988; Hirsh et al, 1988). It is very difficult to evaluate some borderline cases based only on histopathological features. It is hoped that biological tumour markers could be used to complement morphological diagnosis. Several kinds of neuroendocrine markers [e.g. neuron-specific enolase (NSE), gastrin-releasing peptide (GRP), creatine kinase BB, neural cell adhesion molecule (NCAM), etc.] are useful to distinguish small-cell lung cancer from non-small-cell lung cancer (Gazdar et al, 1981; Carney et al, 1982; Maruno et al, 1989; Kibbelaar et al, 1991). Non-small-cell lung cancer is a heterogeneous group of tumours including adenocarcinoma, squamous cell carcinoma and large-cell carcinoma, and each histopathological type of tumour displays a characteristic marker pattern. However, no current tumour markers for non-small-cell lung cancer, including adenocarcinoma, give us satisfaction in terms of either sensitivity or specificity.

In this context, we recently showed using 2-DE the possibility that primary lung carcinoma could be evaluated based on the expression levels of several kinds of unidentified polypeptides associated with histopathological differentiation. (Hirano et al, 1995). We believe that this classification of primary lung carcinoma, based on 2-DE findings, in combination with morphological features reflects the biological features of the tumour more accurately than morphological features alone. TA01 and TA02 polypeptides occupied an important position in our previous analysis because these two polypeptides were representatively associated with primary adenocarcinoma of the lung. Furthermore, TA01 and TA02 polypeptides showed the highest expression levels on 2-DE gels of most WD adenocarcinomas, and metastatic lung adenocarcinomas from the colon and most breast carcinomas did not express either of these two polypeptides. Therefore, in this study, we concentrated on the analysis of the relationships between the expression levels of these two polypeptides and histocytological features of primary adenocarcinoma of the lung.

We developed a non-enzymatic sample preparation technique for removal of serum proteins, necrosis, mesenchymal cells and inflammatory cells (Franzèn et al, 1994; Okuzawa et al, 1994;). This unique sample preparation technique enabled both high-resolutional 2-DE analysis of clinical materials and accurate analysis of cancer proteins. When the lysate of whole tumour tissues was used as samples for 2-DE without using our sample preparation methods, there were many changes in the 2-DE patterns, e.g. increase of expression levels of all tropomyosin isoforms, which normal cells express, and decrease in expression levels of TA01 and TA02 polypeptides. These findings show that cancerous cells of primary adenocarcinoma of the lung themselves produced TA01 and TA02 polypeptides. We think that the decrease in expression levels of TA01 and TA02 polypeptides was because of contamination and dilution by several kinds of normal cells.

Most cases with high expression of TA01 and TA02 polypeptides were diagnosed as WD adenocarcinoma cytologically and histologically. On the other hand, most cases with low expression of these polypeptides showed high cellular and structural atypia and were diagnosed as either PD or MD adenocarcinoma. Furthermore, it was difficult to preoperatively diagnose cytological specimens of some cases with undetectable levels of either TA01 and TA02 polypeptides as adenocarcinomas.

We concluded that TA01 and TA02 polypeptides are related to histopathological differentiation of primary lung adenocarcinoma. Cases with high expression of TA01 and TA02 polypeptides showed a growth pattern resembling bronchioloalveolar cell carcinoma at least in a part of the tumour (especially in peripheral areas of tumour). Although only 2 out of 23 cases diagnosed as WD adenocarcinoma had undetectable levels of both TA01 and TA02 polypeptides, detailed histopathological examination of these two

© Cancer Research Campaign 1997

cases revealed goblet cell type of adenocarcinoma (Figure 3). This type of adenocarcinoma derives from the mucous cells that distribute in relatively peripheral bronchi and not in the alveolar region. With respect to the cells of origin of cancer cells with high expression of TA01 and TA02 polypeptides, it was suggested that these two polypeptides accumulated in type II pneumocytes of the alveolar sac and/or non-ciliated non-mucous cells in the bronchioles (Clara cells) where most primary adenocarcinomas of the lung originate. However, at present, we do not have any direct evidence of the cellular distribution of these two polypeptides in normal tissues because the resolution of 2-DE is very low in normal peripheral lung tissues. Production of antibodies against these polypeptides is needed for further investigation. Recently, we obtained mouse polyclonal antibody against TA02 polypeptide by subcutaneous injection of TA02 polypeptide. According to our preliminary data using this polyclonal antibody, TA02 polypeptide is distributed in the cell membrane and cytoplasm of primary lung adenocarcinomas, in particular WD adenocarcinomas. Also, the cell membrane and cytoplasm of part of the alveolar epithelium (probably type II pneumocyte) revealed positive staining (data not shown). At present, we are trying to produce the monoclonal antibodies for further investigation of these molecules.

It would be reasonable that the distribution of these two polypeptides in cancer tissues compared with that of known polypeptides would resemble that of surfactant apoprotein A, Clara cell-specific 10-kDa protein and protein 1 in primary lung carcinoma (Singh et al, 1988; Auten et al, 1990; Barnard et al, 1992; Linnoila et al, 1992). The molecular weights of Clara cell-specific 10-kDa protein and protein 1 are completely different from those of TA01 and TA02 polypeptides. However, the molecular weight of surfactant apoprotein A (35–37 kDa) is approximately that of TA01 and TA02 polypeptides. Therefore, we attempted to detect TA01 and TA02 polypeptide using anti-surfactant apoprotein A monoclonal antibody (PE-1, DAKO) after 2-DE and Western blotting in cases with high expression of TA01 and TA02 polypeptides. However, these two polypeptides could not be detected by the PE-1 monoclonal antibody (data not shown).

In the cases with high expression of TA01 and TA02 polypeptides, we detected a third polypeptide with the same molecular weight. Its isoelectric point (pI) was approximately 5.24 (Figure 2). These findings show that there is a possibility that the TA02 polypeptide and the third polypeptide may be phosphorylated forms of the TA01 polypeptide.

Finally, our present data indicate that expression levels of TA01 and TA02 polypeptides correlate with the histopathological differentiation of adenocarcinoma. At present, histopathological diagnosis, especially concerning differentiation, is based on subjective observation by experienced pathologists. If we could succeed in producing monoclonal antibodies against TA01 and TA02 polypeptides, quantitative and objective diagnosis of the degree of differentiation of adenocarcinoma may be realized.

### ABBREVIATIONS

CHAPS, 3-[(3-Cholamidopropyl) dimethylammonio]-1-propanesulphonate; 2-DE, two-dimensional polyacrylamide gel electrophoresis; pI, isoelectric point; MD adenocarcinoma, moderately differentiated adenocarcinoma; MW, molecular weight; PMSF, phenylmethyl sulphonyl fluoride; PD adenocarcinoma, poorly differentiated adenocarcinoma; SDS, sodium dodecyl sulphate; WD adenocarcinoma, well-differentiated adenocarcinoma.

### ACKNOWLEDGEMENTS

We would like to thank Ayako Nakagawa for her skillful 2-DE technique. The authors also thank Professor J Patrick Barron of the International Medical Communications Center of Tokyo Medical College for his review of the manuscript.

### REFERENCES

Addis BJ (1988) Pathology of Lung Cancer. In *Lung tumors*, Hoogstraten B, Addis BJ, Hansen HH, Martini N and Spiro SG. (eds), pp. 17–36. Springer: Berlin

Auten RL, Watkis RH, Shapiro DL and Horowitz S (1990) Surfactant apoprotein A (SP-A) is synthesized in air way cells. *Am J Respir Cell Mol Biol* **3**: 491–496

Barnard A, Roels H, Lauwerys R, Gielens C, Soumillion A, Van Damme J and Delay M (1992) Protein 1 is a secretory protein of the respiratory and urogenital tracts identical to the Clara cell protein. *Clin Chem* **38**: 434–435

Bhattacharya B, Prasad GL, Valverius EM, Salomon DS and Cooper HL (1990) Tropomyosins of human mammary epithelial cells: consistent defects of expression in mammary carcinoma cell lines. *Cancer Res* **50**: 2105–2112

Bjellqvist B, Basse B, Olsen E and Celis JE (1994) Reference points human cell types defined in a pH scale where isoelectric points correlate with polypeptide compositions. *Electrophoresis* **15**: 529–539

Buccheri G and Ferrigno D (1988) Usefulness of tissue polypeptide antigen in staging, monitoring and prognosis of lung cancer. *Chest* **93**: 565–570

Carney DN, Ihde DC, Cohen MH, Bunn Jr PA, Cohen MH and Minna JD (1982) Serum neuron-specific enolase. A marker for disease extent and response to therapy of small-cell lung cancer. *Lancet* **1**: 583–585

Celis JE, Rasmussen HH, Madsen P, Leffers H, Honore B, Dejgaarg K, Gesser B, Olsen E, Gromov P, Hoffmann H, Nielsen M, Celis A, Basse B, Lauridsen JB, Ratz GP, Nielsen H, Andersen AH, Walbum E, KJ Rgaard I, Puype M, Damme JV and Vandkerckhove J (1992) The human keratinocyte two-dimensional gel protein database. *Electrophoresis* **13**: 893–959

Costa A, Silvestrini R, Mochen C, Lequaglie C, Boracchi P, Faranda A, Vessecchia G and Ravasi G (1996) p53 expression, DNA ploidy and S-phase cell fraction in operable locally advanced non-small cell lung cancer. *Br J Cancer* **73**: 914–919

Ebina M, Steinberg SM, Mulshine JL and Linnoila RI (1994) Relationship of p53 overexpression and up-regulation of proliferating cell nuclear antigen with the clinical course of non-small cell lung cancer. *Cancer Res* **54**: 2496–2503

Franzèn B, Okuzawa K, Linder S, Kato H and Auer G (1994) Non-enzymatic extraction of cells from clinical tumor material for analysis of gene expression by two-dimensional polyacrylamide gel electrophoresis. *Electrophoresis* **14**: 1045–1053

Garrels JI and Franza Jr BR (1989) Transformation-sensitive and growth-related changes of protein synthesis in REF52 cells. *J Biol Chem* **264**: 5299–5312

Gazdar AF and Linnoila RI (1988) The pathology of the lung. Changing concepta and newer diagnostic techniques. *Semin Oncol* **15**: 215–225

Gazdar AF, Zweig MH, Carney DN, Van Steirtehgen AC, Baylin SB and Minna JD (1981) Levels of creatine kinase and its BB isoenzyme in lung cancer tumor and cultures. *Cancer Res* **41**: 2773–2777

Hirano T, Franzèn B, Uryu K, Okuzawa K, Alaiya AA, Vanky F, Rodrigues Y, Ebihara Y, Kato H and Auer G (1995) Detection of polypeptides associated with the histopathological differentiation of primary lung carcinoma. *Br J Cancer* **72**: 840–848

Hirsh FA, Matthews MJ, Aisner S, Campobasso O, Elema JD, Gazdar AF, Mackay B, Nasiell M, Shimosato Y, Steele RH, Yesner R and Zettergren L (1988) Histopathological classification of small cell lung cancer. *Cancer* **62**: 973–977

Kibbelaar RE, Moolenaar KEC, Michalides RJAM, Van Bodegon PC, Vanderschueren RGJRA, Wagenaar SS, Dingemans KP, Bitter-Suermann D, Dalesio O, Dalesio O, Van Zandwijk N and Mooi WJ (1991) Neural cell adhesion molecule expression, neuroendocrine differentiation and prognosis in lung carcinoma. *Eur J Cancer* **27**: 431–435

Latanche G, Ogier I and Weynants P (1985) Diagnostic value of the assay of carbohydrate antigen 19–9 (CA 19–9) in patients with primary bronchial cancer. *Rev Pneumol Clin* **41**: 314–316

Linnoila RL, Jensen SM, Steinberg SM, Mulshine JL, Egglestone JC and Gazdar AF (1992) Peripheral airway cell marker expression in non-small cell lung carcinoma. *Am J Clin Pathol* **97**: 233–243

Maruno K, Yamaguchi K, Abe K, Suzuki M, Saijo N, Mishima Y, Yanaihara N and Shimosato Y (1989) Immunoreactive gastrin-releasing peptide as a specific tumor marker in patients with small cell lung cancer. *Cancer Res* **49**: 629–632

 © Cancer Research Campaign 1997

Mørkve O, Halvorsen OJ, Skjaerven R, Stangeland L, Gulsvik A and Laerum OD (1993) Quantitation of biological tumor markers (p53, c-*myc*, Ki-67 and DNA ploidy) by multiparameter flow cytometry in non-small cell lung cancer. *Int J Cancer* **52**: 851–855

Morrissey JH (1981) Silver stain for proteins in polyacrylamide gels: a modified procedure with enhanced uniform sensitivity. *Anal Biochem* **117**: 307–310

Mountain CF (1986) A new international staging system for lung cancer. *Chest* **89**: 225–232

Niklinski J and Furman M (1995) Clinical tumour markers in lung cancer. *Eur J Cancer Prev* **4**: 129–138

Nomori H, Shimosato Y, Kodama T, Morinaga S, Nakajima T and Watanabe S (1986) Subtypes of small cell carcinoma of the lung: morphometric, ultrastructural and immunohistochemical analysis. *Hum Pathol* **17**: 604–613

Ochs DC, McConkey EH and Guard NL (1981) Vimentin-derived proteins. *Exp Cell Res* **135**: 355–362

O'Farrell PH (1975) High resolution two-dimensional electrophoresis of proteins. *J Biol Chem* **250**: 4007–4021

Okuzawa K, Franzèn B, Lindholm J, Linder S, Horano T, Bergman T, Ebihara Y, Kato H and Auer G (1994) Characterization of gene expression in clinical lung materials by two-dimensional polyacrylamide gel electrophoresis. *Electrophoresis* **15**: 382–390

Rachwal WJ, Bongiorno PF, Orringer MB, Whyte RI, Ethier SP and Beer DG (1995) Expression and activation of *erb*B-2 and epidermal growth factor receptor in lung adenocarcinomas. *Br J Cancer* **72**: 56–64

Radice PA, Forest N, Ihde DC, Gazdar AF, Carney DN, Bunn PA, Cohen MH, Fossieck BE, Makuch RW and Minna JD (1982) The clinical behaviour of 'mixed' small cell/large cell bronchogenic carcinoma compared to 'pure' small cell subtypes. *Cancer* **50**: 2894–2902

Scagliotti GV, Micela M, Gubetta L, Leonardo E, Cappia S, Borasio P and Pozzi E (1993) Prognostic significance of Ki67 labelling in resected non-small cell lung cancer. *Eur J Cancer* **29A**: 363–365

Singh G, Singh J, Katyal SL, Dauber JH, MacPherson TA and Squeglia H (1988) Identification, cellular localization, isolation and characterization of human Clara cell-specific 10kDa protein. *J Histochem Cytochem* **36**: 73–80

Vincent RG, Chu TM and Lane WW (1979) The value of carcinoembryonic antigen in patients with carcinoma of the lung. *Cancer* **44**: 685–691

WHO (1992) The World Health Organization histological typing of lung tumours, 2nd edn. *Am J Clin Pathol* **77**: 123–136

© Cancer Research Campaign 1997